# Chinese herbal medicine Yanghe decoction for knee osteoarthritis: A protocol for systematic review and meta-analysis

**DOI:** 10.1097/MD.0000000000021877

**Published:** 2020-08-21

**Authors:** Xiaosheng Xu, Yi Wan, Liangjin Gong, Zeren Ma, Tao Xu

**Affiliations:** Nanchang Hongdu Hospital of Traditional Chinese Medicine, Nanchang, China.

**Keywords:** knee osteoarthritis, protocol, systematic review, Yanghe decoction

## Abstract

**Background:**

Chinese herbal medicine (CHM) has been widely used to treat knee osteoarthritis (KOA), among which Yanghe decoction (YHD) is one of the commonly used prescriptions. The purpose of this study is to evaluate the effectiveness and safety of YHD in the treatment of KOA.

**Methods:**

Six databases, including Embase, PubMed, the Cochrane Library, the China National Knowledge Infrastructure, Wanfang Database and Chinese Science and Technology Periodical Database will be searched from their inception to July 2020. Two researchers will independently select studies, collect data and evaluate the quality of included studies. Statistical analysis will be processed by RevMan V.5.3 software.

**Results:**

This study will provide an assessment of the current state of YHD in the treatment of KOA, aiming to show the efficacy and safety of YHD.

**Conclusion:**

This study will provide evidence to judge whether YHD is an effective intervention for KOA.

## 1 Introduction

Knee osteoarthritis (KOA) is one of the most common chronic diseases and the main cause of disability worldwide.^[1,2]^ KOA is characterized by joint degeneration leading to pain, limited motor function, muscle weakness, joint stiffness and swelling.^[3]^ According to statistics, the incidence of KOA among the elderly is relatively high, and approximately 30% to 50% of the elderly over 60 are affected by KOA.^[4]^ Due to its progressive and chronic symptoms, KOA not only reduces the quality of life of the affected individuals, but also requires a large amount of healthcare resources and involves considerable social treatment costs, and these needs will inevitably increase with the aging of the population.^[5,6]^

Regarding the treatment management of KOA, the current treatment methods for KOA are mainly aimed at reducing joint pain and slowing down its progress. The International Osteoarthritis Society (OARSI) recommends conservative treatment as the first-line treatment for KOA.^[7]^ Among the conservative treatment methods, drug treatment is a widely used method in clinical practice and recommended by clinical guidelines.^[7,8]^ Although drug therapy can alleviate the symptoms of KOA, its long-term application is usually accompanied by certain side effects.^[9,10]^ Therefore, it is necessary to find a more effective and safe alternative therapy to treat KOA. Chinese herbal medicine (CHM) is one of the widely used alternative therapies around the world, and recent systematic reviews have shown that CHM is effective for the treatment of KOA.^[11,12]^

Yanghe decoction (YHD) is a well-known prescription, which is composed of rehmannia, deerhorn glue, cinnamomum cassia, semen brassicae, ephedra, charcoal of ginger and radix glycyrrhizae.^[13]^ Modern pharmacological research shows that YHD has the effect of preventing chondrocyte apoptosis and anti-inflammatory during the treatment of KOA.^[14,15]^ Therefore, YHD is regarded as a safe and effective alternative therapy.

Although YHD has been widely used in the treatment of KOA,^[16,17]^ there is no systematic review to explore the effectiveness of YHD on KOA. Therefore, the purpose of this study is to evaluate the effectiveness and safety of YHD for KOA based on current studies.

## 2 Methods

This study has been registered with the Open Science Framework (OSF, https://osf.io/). The registration DOI of this protocol is 10.17605/OSF.IO/X7VDP. We will follow the preferred reporting items for the systematic review and meta-analysis (PRISMA) to perform this study.

### 2.1 Inclusion criteria for study selection

#### 2.1.1 Types of studies

RCTs evaluating YHD treatment for KOA will meet the criteria for inclusion. Observational studies and non-RCTs will be excluded.

#### 2.1.2 Types of participants

We will consider KOA patients with a clear diagnosis, without gender, race, or age restrictions.

#### 2.1.3 Types of interventions

##### 2.1.3.1 Experimental interventions

The intervention method of the experimental group is YHD or modified YHD, and there is no limit to the type of administration, dose or treatment time.

##### 2.1.3.2 Control interventions

The treatment of the control group will include drug treatment, no treatment and placebo.

#### 2.1.4 Types of outcome measures.

##### 2.1.4.1 Primary outcome

Visual analogue scale (VAS) and Clinical efficacy will be regarded as the primary outcomes.

##### 2.1.4.2 Additional outcomes

The safety assessment will be accepted as an additional result.

### 2.2 Search methods for the identification of studies

The following databases will be searched: PubMed, Embase, Cochrane Library, the China National Knowledge Infrastructure, Chinese Science and Technology Periodical Database and Wanfang Database. We will search the databases from the beginning to July 2020. Search terms consist of disease (knee osteoarthritis OR knee osteoarthritides OR knee pain OR knee joint osteoarthritis OR knee arthritis OR osteoarthritis of knee) and intervention (Yanghe decoction OR Yanghe Tang) and research types (randomized controlled trial, controlled clinical trial, random trials). The PubMed search strategy is shown in Table 1.

**Table 1:**
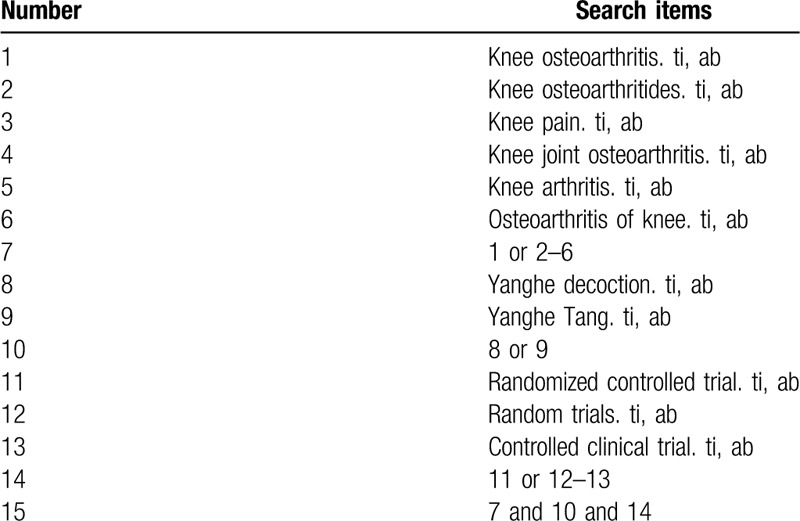
Search strategy used in PubMed database.

### 2.3 Data collection and analysis

#### 2.3.1 Selection of studies

**Figure 1:**
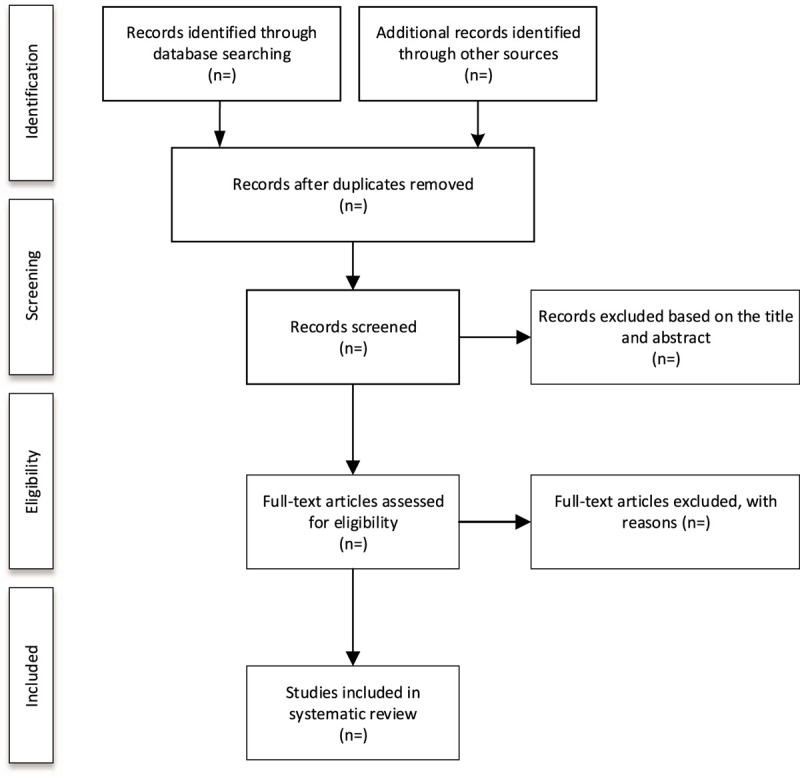
Flow diagram of study selection process.

Two authors will independently search the literature based on the formulated criteria, and import the citations retrieved from the database into EndNote X9 software for management, and then evaluate the eligibility of these articles based on the inclusion criteria. When there is a disagreement, the two authors will discuss and negotiate with the third author. The study selection procedure is summarized in Figure 1.

#### 2.3.2 Data extraction and management

The information extraction of the included studies will be completed by two researchers independently, and any disagreement will be resolved through discussion with a third researcher. The extracted information will mainly include the first author, year of publication, study design, participant characteristics, sample size, interventions, results, and adverse events.

### 2.4 Risk of bias assessment

Two independent researchers will evaluate the risk of bias based on the Cochrane Collaboration's tool. Any disagreements will be resolved through discussion. The tool has 7 domains, which include random sequence generation, allocation concealment, the blinding method for patients, researchers and outcomes assessors, incomplete result data, selective reports and other sources of bias. Each item will be evaluated as high, unclear or low risk of bias.^[18]^

### 2.5 Quantitative data synthesis and statistical methods

#### 2.5.1 Quantitative data synthesis

Statistical analysis will be carried out using RevMan 5.3 software. Continuous data will be calculated as mean difference (MD) with 95% CI, and dichotomous data will be calculated as risk ratio (RR) with 95% CI.

#### 2.5.2 Assessment of heterogeneity

We will use chi-square test and *I*^2^ test to assess heterogeneity. When *P* > .10 and *I*^2^ ≤ 50%, it indicates that there is no heterogeneity or the heterogeneity is not obvious; on the contrary, when *P* < .10 or *I*^2^ > 50%, it indicates that the study has significant heterogeneity.

#### 2.5.3 Assessment of reporting biases

If there are no less than 10 RCTs included in this study, we will use a funnel plots to evaluate publication bias. Otherwise, we will evaluate by Egger test.

#### 2.5.4 Subgroup analysis

If there is significant heterogeneity in the included studies, we will conduct subgroup analysis according to the type of control group.

#### 2.5.5 Sensitivity analysis

If sufficient RCTs are available for our research, we will conduct a sensitivity analysis to assess the robustness and reliability of the meta-analysis by eliminating low-quality studies.

#### 2.5.6 Grading the quality of evidence

We will assess the quality of evidence by the Grading of Recommendations Assessment, Development and Evaluation and rate it into high, medium, low or very low 4 levels.^[19,20]^

## 3 Discussion

CHM has been widely used in the treatment of KOA, and YHD has always been one of the commonly used prescriptions for KOA treatment. Although previous studies have reported the benefits of YHD treatment for KOA,^[16]^ the effectiveness of YHD still lacks comprehensive systematic reviews and research evidence. The purpose of this study is to evaluate the effectiveness and safety of YHD in the treatment of KOA. The conclusions of this study will provide evidence-based medicine advice for YHD treatment of KOA.

## Author contributions

**Data curation:** Xiaosheng Xu, Yi Wan.

**Formal analysis:** Xiaosheng Xu, Yi Wan.

**Investigation:** Yi Wan, Liangjin Gong.

**Methodology:** Yi Wan, Liangjin Gong.

**Project administration:** Zeren Ma, Tao Xu.

**Software:** Yi Wan, Liangjin Gong.

**Supervision:** Tao Xu.

**Validation:** Tao Xu, Liangjin Gong.

**Visualization:** Zeren Ma, Liangjin Gong.

**Writing – original draft:** Xiaosheng Xu, Tao Xu.

**Writing – review & editing:** Xiaosheng Xu, Tao Xu.
